# Developmental mentoring in otolaryngology: a qualitative study highlighting gaps in understanding and implementation

**DOI:** 10.1017/S0022215126105428

**Published:** 2026-09

**Authors:** Emma Stapleton, Rohma Abrar, Sirat Lodhi, Women in ENT UK

**Affiliations:** 1 Manchester Academic Health Sciences Centre, The University of Manchester Faculty of Biology Medicine and Health, UK; 2 Department of Otolaryngology, Manchester Royal Infirmary, UK; 3 Manchester Royal Infirmary, UK; 4 Manchester University NHS Foundation Trust, UK; 5 WENTS ENT UK, UK

**Keywords:** education, medical, mentoring, otolaryngology

## Abstract

**Introduction:**

Developmental mentoring is widely advocated within Otolaryngology training, yet programmes are often poorly defined and may not reflect its intended distinction from traditional hierarchical models. This study explored participant understanding of mentoring within a national Otolaryngology mentoring programme.

**Methods:**

Prospective participants completed open-ended questionnaires prior to mentor training. Responses were analysed using grounded theory with iterative coding and constant comparison.

**Results:**

Twenty-seven participants responded. Many identified features consistent with developmental mentoring, including empowerment, goal exploration, reflection and psychological safety. However, there was notable variability in interpretation; some uncertain of the concept, others describing supervision or patronage. Broader personal, professional and value-based elements relevant to Otolaryngology training were also emphasised.

**Conclusion:**

Understanding of developmental mentoring is heterogeneous and often diverges from its intended model. This lack of shared understanding may undermine programme effectiveness and efforts to support the Otolaryngology workforce. Clear definition and participant training are essential for successful implementation.

## Introduction

Mentoring is widely advocated within surgical training and is associated with improved career progression, satisfaction and retention.^1^^–^^3^ In Otolaryngology, national mentoring initiatives have been developed to support trainees and promote a more inclusive workforce.^4^^,^^5^ Previous work has demonstrated that participants entering such programmes hold diverse expectations regarding mentoring relationships, highlighting early variability in understanding.^6^

Traditional models of supervision or patronage are typically hierarchical, with a senior clinician providing guidance and support to a more junior colleague.^7^^–^^9^ In contrast, developmental mentoring emphasises a collaborative, non-hierarchical relationship in which the mentor facilitates reflection and supports the mentee in defining and achieving their own goals.^10^^–^^12^

Despite this distinction, mentoring programmes are frequently implemented without clear definition or shared understanding of the model being used.^13^^,^^14^ This lack of clarity risks mentoring programmes being established in name, but not in practice. Participants may therefore enter mentoring relationships with differing expectations, potentially limiting programme effectiveness and reproducing outdated dynamics of hierarchical supervision, patronage or sponsorship.

Furthermore, the sustainability of mentoring programmes depends on their perceived value and effectiveness. Programmes that lack clarity in purpose, structure and participant understanding may fail to achieve meaningful impact, risking disengagement and limiting long-term viability.^15^^,^^16^

Although mentoring is well described within medical education, there is limited understanding of how developmental mentoring is interpreted by participants within surgical training, particularly in Otolaryngology.

We therefore conducted a qualitative study within a national Otolaryngology mentoring programme to explore participant understanding of developmental mentoring prior to formal training.

## Methods

### Participant recruitment

Women in ENT Surgery (WENTS) is a national organisation endorsed by ENT UK and established the first national mentoring programme in Otolaryngology, open to Otolaryngologists of all genders and backgrounds.

Prior to embarking on their formal mentor training to participate in the programme, a cohort of prospective mentors and mentees were invited via email to participate in this study, by responding to the open-ended question: *“What is your understanding of developmental mentoring in surgery?”* Participants were encouraged to provide detailed responses. All participants received a participant information sheet and were given the opportunity to ask questions before providing informed consent. Participation was voluntary, and participants were free to withdraw at any time. The Health Research Authority decision tool confirmed that National Health Service (NHS) Research Ethics Committee approval was not required.

### Data analysis

Responses were analysed using a grounded theory approach. Initial coding was undertaken independently by the three main authors, with codes developed iteratively using a constant comparison technique. Codes were discussed and refined through consensus to develop a coding framework and were subsequently grouped into categories and overarching themes.

Analysis was conducted in iterative cycles to ensure that emerging concepts were explored and refined across the dataset. Data saturation was reached after 24 responses; however, all responses were analysed and coded. Dedoose qualitative analysis software (Los Angeles, CA, USA) was used to support data organisation and analysis.

## Results

Twenty-seven participants responded to the study question. Three were medical students, nine were junior doctors (foundation and core surgical trainees), 12 were specialty trainees and three were consultants. Thirteen participants were mentees only, four were mentors only and 10 undertook dual roles.

While most participants were able to describe key elements of developmental mentoring, there was notable variability in interpretation. Four participants reported uncertainty regarding the concept, and several descriptions aligned more closely with traditional, directive mentoring models such as supervision, patronage and sponsorship. This variability was evident across all training grades.

Six key themes were identified. Four reflected recognised aspects of developmental mentoring: (1) empowering the mentee, (2) exploring mentee goals and expectations, (3) use of reflection from personal experience and (4) maintaining a safe mentoring space without conflict of interest (Table 1). Two additional themes were identified: (5) mentoring domains encompassing personal and professional factors and (6) key values and ethos. These are expanded below and in Tables 1–3, with sample quotes.

**Table 1.** Key aspects of developmental mentoring techniques in surgery Table 1 long description.

Theme 1 - Empower the mentee	- *Empower* the mentee to take control of their own development
	- *Nurture* the mentee to help them grow independently & increase their confidence
	- *Signpost* developmental opportunities to facilitate the mentee
	- Recognise *individuality* in mentees of their values, morals and career paths, rather than dictating your path to them
Theme 2 - Explore mentee goals & expectations	- Explore *mentee goals, motivations and expectations* using active listening
	- Plan the future by *setting mentee goals together* based on mentee motivations
	- Maintain a positive focus
Theme 3 - Use reflections from personal experiences to guide the mentee	*- Share own experiences* such as difficult cases to enable learning points to be identified that the mentee can gain from
	- Impart your wisdom and what “*you wish you had known”* when you were at the mentee’s stage
	- *Encourage reflection from the mentee* to enable the mentee to better realise the path they wish to take
Theme 4 - Create a safe mentorship space without conflict of interest	- Ideal for mentor to be outside the mentee’s department:
	o to avoid *personal or professional conflict of interest* between the mentor and mentee
	o to encourage open discussion by *removing the direct line of accountability*
	o to enable mentor to offer *unbiased and objective advice* and perspective on mentee’s issues

### Theme 1: Empower the mentee

Participants frequently and accurately described developmental mentoring as a process of enabling the mentee to take ownership of their development. This included supporting independence, confidence and self-direction:

“To empower the mentee to seek the answers they need for themselves… instead of dictating a path” (P22)

Participants emphasised guiding rather than directing, with a focus on nurturing growth and facilitating opportunities rather than providing them directly. Several described the role of signposting development opportunities and supporting the mentee to recognise their own capabilities.

### Theme 2: Explore mentee goals and expectations

Exploration of mentee goals and motivations was closely linked to empowerment. Participants highlighted the importance of open discussion to define objectives and develop realistic plans:

“Helping mentees… by exploring their goals and motivations” (P24)

Mentoring was described as a collaborative process in which mentor and mentee work together to clarify direction and define achievable outcomes.

### Theme 3: Use of reflection from personal experience

Participants described the use of reflective practice as central to developmental mentoring. Mentor experiences were viewed as a tool to facilitate mentee reflection and learning:

“Using their experiences to help enable a mentee to reflect and develop their own personal goals” (P8)

Importantly, responsibility for development was consistently attributed to the mentee, with the mentor acting as a guide rather than a director.

### Theme 4: Safe mentoring space without conflict of interest

A key theme was the importance of a psychologically safe environment, free from hierarchical pressures or conflicts of interest. Participants emphasised the value of mentors being external to the mentee’s immediate clinical environment:

“Not being directly involved… thus offering an objective and unbiased perspective” (P15)

This was perceived to facilitate open discussion and enable more honest reflection.

#### Theme 5: Mentoring domains: personal and professional factors

Participants identified a broad range of areas encompassed within developmental mentoring (Table 2), including career progression, clinical and research development and training opportunities. Pastoral elements were also emphasised, including work–life balance, interpersonal relationships and wellbeing:

“As much, if not more, to do with… work/life balance… and emotional wellbeing” (P20)

**Table 2.** Theme 5: mentoring areas Table 2 long description.

*Professional support*
- Career choices
- Portfolio development
- Clinical skills
- Training & development opportunities
*Pastoral support*
- Work-life balance
- Interpersonal relationships with colleagues
- Personal wellbeing

### Theme 6: Key values and ethos

Participants described core values underpinning developmental mentoring, including inclusivity, trust, honesty, confidentiality and mutual respect (Table 3). Adaptability over time was also emphasised:

“The relationship should be based on trust… matched by the enthusiasm of the mentee” (P14)

**Table 3.** Theme 6: developmental mentoring ethos & values Table 3 long description.

- *Inclusivity and fairness*
- *Openness*
- *Honesty*
- *Maintaining confidentiality*
- *Professionalism*
- *Non-judgemental attitude*
- *Respect*
- *Adaptability*
- *Trust*
- *Generosity from mentor*
- *Enthusiasm from mentee*

## Discussion

This study demonstrates that, while participants in a national Otolaryngology mentoring programme can largely identify key elements of developmental mentoring, there is substantial variability in how the concept is understood. This variability was evident across all training grades and included both uncertainty and interpretations aligned with traditional hierarchical models such as supervision, patronage and sponsorship. These can confuse or dilute developmental mentoring in its true form.

This has important implications for the implementation of mentoring programmes. Although developmental mentoring is increasingly advocated within surgical training, our findings suggest that it cannot be assumed to be intuitively understood by participants. Without a shared conceptual framework, mentoring relationships may be established with differing expectations, risking misalignment between mentor and mentee and limiting programme effectiveness. In this context, mentoring programmes may exist in name, but not in practice.

The themes identified broadly align with established principles of developmental mentoring, including empowerment, exploration of goals, reflective practice and the creation of a psychologically safe space.^10^^–^^12^ However, their coexistence with more directive interpretations highlights a lack of clarity in how developmental mentoring is conceptualised in practice. Participants appear to draw on multiple models of mentoring simultaneously, rather than engaging with a clearly defined approach.

A key implication of this finding is its impact on programme effectiveness and sustainability. Mentoring programmes rely on participant engagement, perceived value and the development of meaningful relationships. Where expectations are misaligned, or interactions default to traditional hierarchical dynamics, the intended benefits of developmental mentoring may not be realised. Over time, this may limit engagement and undermine programme sustainability.^15^^,^^16^

These findings build on previous work demonstrating variability in participant expectations within otolaryngology mentoring programmes,^6^ extending this to show that variability exists at the level of conceptual understanding. This reinforces the need for explicit definition and preparation prior to programme participation.

Participants also emphasised the importance of pastoral support, inclusivity and values such as trust and confidentiality, reflecting the broader role mentoring is perceived to play within surgical training. While consistent with existing literature, this further highlights the need to define the scope and boundaries of developmental mentoring to avoid overlap with supervision or pastoral roles.

Taken together, these findings suggest that effective implementation of developmental mentoring requires more than programme establishment alone. Clear articulation of the mentoring model, structured training for participants and alignment of expectations are essential to ensure that mentoring relationships reflect developmental principles rather than defaulting to traditional hierarchical approaches.

- Mentoring is widely advocated within Otolaryngology training and is associated with improved career development, satisfaction and retention
- Developmental mentoring differs from traditional hierarchical models, emphasising a collaborative, non-directive approach
- Mentoring programmes are increasingly established, but are often implemented without clear definition or understanding of the underlying model
- This study demonstrates substantial variability in participant understanding of developmental mentoring, including uncertainty and overlap with traditional models such as supervision and patronage
- Variability in understanding is evident across all training grades and roles within a national otolaryngology mentoring programme
- Lack of a shared conceptual framework may limit programme effectiveness and sustainability, highlighting the need for clear definition and structured participant training

## Conclusion

Understanding of developmental mentoring within Otolaryngology is heterogeneous and frequently diverges from its intended conceptual model. While participants recognise key elements of developmental mentoring, variability in interpretation and overlap with traditional hierarchical models may limit the effectiveness of mentoring programmes.

For mentoring programmes to achieve their intended impact, clear definition of developmental mentoring, structured participant training and alignment of expectations are essential. Without this, there is a risk that programmes are implemented without delivering meaningful benefit, with implications for both sustainability and workforce development in our specialty.

## Data Availability

Primary data are available by request.

## WENTS National Mentoring Collaborative authors

Anastasha Herman, Dorota Chudek, E Tian Tan, Eniola Salau, Hannah Lancer, Lauren Bolton, Martina Spazzapan, Megan E. Walker, Natalie A. Watson, Olivia Kenyon, Paula Coyle, Rania Fernandes, Rujuta Roplekar, Sevasti Konstantinidou, Yasmin Abbas, Yasmin Nikookam.

## Table 1 Long description

Four themes outline developmental mentoring techniques in surgery. Theme one emphasizes empowering mentees to direct their own development by nurturing independence and confidence, signposting opportunities, and respecting individual values and career paths. Theme two focuses on exploring goals, motivations, and expectations through active listening, then co-setting future goals while maintaining a positive focus. Theme three highlights using mentors’ personal experiences, including difficult cases and lessons they wish they had known, while encouraging mentee reflection to clarify desired paths. Theme four stresses creating a safe mentorship space by choosing a mentor outside the mentee’s department to reduce conflicts of interest, remove direct accountability pressures, and support open discussion with unbiased advice. The themes are qualitative categories rather than ranked or quantified outcomes.

Navigate back to Table 1.

## Table 2 Long description

Mentoring areas are listed as categories of support that mentoring can cover. The entries include professional support, career choices, and portfolio development. Skills and growth topics are represented by clinical skills and training and development opportunities. Personal and relational support areas include pastoral support, work-life balance, interpersonal relationships with colleagues, and personal wellbeing. No counts, ratings, or rankings are provided, so the list indicates scope rather than relative priority or frequency.

Navigate back to Table 2.

## Table 3 Long description

Developmental mentoring ethos is represented as a list of values rather than numeric results. Values named include inclusivity and fairness, openness, honesty, and maintaining confidentiality. Additional qualities are professionalism, a non-judgemental attitude, respect, adaptability, and trust. The list also highlights relational contributions: generosity from the mentor and enthusiasm from the mentee. No rankings, frequencies, or comparisons are provided, so relative importance and prevalence cannot be inferred from the order shown.

Navigate back to Table 3.
